# Supplementary material for: Long-Term Effects and Potential Impact of Early Nutrition with Breast Milk or Infant Formula on Glucose Homeostasis Control in Healthy Children at 6 Years Old: A Follow-Up from the COGNIS Study
Source: Nutrients. 2023 Feb 7;15(4):852. doi: 10.3390/nu15040852 (PMC9965004; doi:10.3390/nu15040852)
Supplement: Supplementary file 1 [file nutrients-15-00852-s001.zip › nutrients-2185483-supplementary.pdf]

Supplementary Materials

Table S1. (Methodology): Dietary Reference Intakes (DRIs) at 6 years old.

Dietary Reference Intakes (DRIs) (38, 40) at 6 years old			
**Total kcal/day adequacy to DRI**	Boys	Deficit	<1709
		Adequate	1709–2064
		Excess	>2064
	Girls	Deficit	<1598
		Adequate	1598–1824
		Excess	>1824
**kcal/kg/day adequacy to DRI**	Boys	Deficit	<84
		Adequate	84–98
		Excess	>98
	Girls	Deficit	<74
		Adequate	74–95
		Excess	>95
**Protein AMDR (%) adequacy to DRI**		Deficit	<10
		Adequate	10–30
		Excess	>30
**Grams of protein/kg/day adequacy to FAO recommendations**		Deficit	<0.72
		Adequate	0.72–0.89
		Excess	>0.89
**CHs AMDR (%) adequacy to DRI**		Deficit	<45
		Adequate	45–65
		Excess	>65
**Simple Sugars AMDR (%) adequacy to DRI**		Adequate	≤25
		Excess	>25
**Lipids AMDR (%) adequacy to DRI**		Deficit	<25
		Adequate	25–35
		Excess	>35
**Linoleic acid (g/day) adequacy to DRI**		Deficit	<10
		Adequate	10
		Excess	>10
**Linolenic Acid (g/day) adequacy to DRI**		Deficit	<0.90
		Adequate	0.90
		Excess	>0.90
**n-6 PUFAs AMDR (%) adequacy to DRI**		Deficit	<5
		Adequate	5–10
		Excess	>10
**n-3 PUFAs AMDR (%) adequacy to DRI**		Deficit	<0.60
		Adequate	0.60–1.20
		Excess	>1.20

AMDR: Acceptable Macronutrient Distribution Ranges; CHs: carbohydrates; DRI: dietary reference intake; FAO: the Food and Agriculture Organization of the United Nations (38); kcal: kilocalories; n-3 PUFAs: omega 3 polyunsaturated fatty acids; n-6 PUFAs: omega 6 polyunsaturated fatty acids.

Table S2. MSE analysis by study group in children at 6 years old with excess weight (EW), calculated using the Slaughter's equations.

Minutes	SF (*n*=8)	EF (*n*=7)	BF (*n*=7)	*p* [1]
MSE 3′	0.200 ± 0.064	0.222 ± 0.047	0.193 ± 0.032	0.82
MSE 6′	0.251 ± 0.078	0.278 ± 0.057	0.242 ± 0.042	0.82
MSE 9′	0.283 ± 0.095	0.316 ± 0.080	0.277 ± 0.056	0.92
MSE 12′	0.315 ± 0.106	0.352 ± 0.099	0.308 ± 0.066	0.91
MSE 15′	0.344 ± 0.119	0.381 ± 0.108	0.318 ± 0.082	0.67
MSE 18′	0.365 ± 0.123	0.415 ± 0.119	0.337 ± 0.107	0.68
MSE 21′	0.371 ± 0.125	0.441 ± 0.140	0.355 ± 0.114	0.85
MSE 24′	0.408 ± 0.142	0.473 ± 0.163	0.370 ± 0.110	0.64
MSE 27′	0.436 ± 0.149	0.472 ± 0.159	0.388 ± 0.146	0.57
MSE 30′	0.446 ± 0.155	0.491 ± 0.163	0.407 ± 0.125	0.64

Data are presented as mean ± SD. [1] p values for overall differences between study groups. ANOVA was carried out. MSE does not have any measurement units. BF: breastfeeding; EF: experimental infant formula; MSE: Multiscale sample entropy; SF: standard infant formula.

Table S3. Children's dietary intake and its adequacy to nutritional recommendations at 6 years old.

Nutrient		6 Years old SF (*n*=30)	EF (*n*=29)	BF (*n*=27)	p^1	p_{adj}^2
Total kcal/day		1653.03 ± 324.36	1740.93 ± 272.28	1663.70 ± 247.75	0.44	0.30
TDEE (kcal/day)		1755.98 ±135.60	1778.97± 165.00	1758.52 ± 147.21	0.82	0.61
Calculated kcal (kcal/day)		1627.47 ±321.44	1711.69± 270.11	1634.70 ± 246.77	0.46	0.31
kcal/kg/day		74.55 ± 19.70	76.68 ± 16.75	72.34 ± 13.83	0.64	0.57
Total protein (g/day)		66.02 ±16.53	69.31 ± 12.06	63.75 ± 12.40	0.32	0.16
Protein AMDR (%)		16.21 ±2.11	16.27 ± 1.99	15.58 ± 1.72	0.35	0.22
Grams of protein/kg/day		2.99 ± 0.79	3.08 ± 0.62	2.82 ± 0.64	0.37	0.33
Total CHs (g/day)		182.73 ± 34.44	181.76 ± 30.39	182.89 ± 37.93	0.99	0.98
CHs AMDR (%)		45.32 ± 5.34	42.77 ± 5.48	44.63 ± 4.52	0.15	0.17
Simple sugars (g/day)		89.27 ± 14.77	83.57 ± 18.86	92.55 ± 20.28	0.17	0.19
Simple sugars AMDR (%) *		22.57 ± 5.21 [a,b]	19.84 ± 4.63 [a]	22.75 ± 4.40 [b]	**0.040**	**0.017**
Total lipids (g/day)		70.27 ± 19.39	78.60 ± 19.60	72.01 ± 10.94	0.16	0.11
Lipids AMDR (%)		38.47 ± 5.00	40.96 ± 5.42	39.78 ± 4.08	0.15	0.17
SFAs (g/day)		25.58 ± 7.04	27.85 ± 7.48	25.87 ± 5.47	0.38	0.29
MUFAs (g/day)		30.12 ± 9.36	34.29 ± 10.02	30.30 ± 5.51	0.12	0.094
PUFAs (g/day)		8.43 ± 2.68	9.59 ± 3.60	9.12 ± 2.48	0.35	0.37
Linoleic acid (g/day)		6.79 ± 2.33	7.47 ± 2.77	7.06 ± 2.03	0.55	0.55
Linolenic acid (g/day)		0.79 ± 0.35	0.84 ± 0.27	0.76 ± 0.18	0.34	0.41
ARA (g/day)		0.11 ± 0.06	0.12 ± 0.07	0.10 ± 0.05	0.55	0.30
EPA (g/day)		0.03 ± 0.04 [a]	0.14 ± 0.23 [b]	0.11 ± 0.17 [a,b]	**0.006**	0.057
DPA (g/day)		0.03 ± 0.03	0.04 ± 0.05	0.03 ± 0.04	0.64	0.53
DHA (g/day)		0.13 ± 0.10 [a]	0.30 ± 0.34[b]	0.25 ± 0.26[a,b]	**0.008**	0.053
n-6 PUFAs (g/day)		5.81 ± 2.11	6.79 ± 2.51	6.36 ± 1.93	0.23	0.25
n-6 PUFAs AMDR (%)		3.17 ± 0.80	3.52 ± 1.03	3.54 ± 1.09	0.27	0.37
n-3 PUFAs (g/day)		0.77 ± 0.38	0.90 ± 0.34	0.75 ± 0.25	0.19	0.11
n-3 PUFAs AMDR (%)		0.43 ± 0.18	0.48 ± 0.19	0.42 ± 0.13	0.25	0.42
Total kcal/day adequacy to DRI boys	Deficit	12 (63.20%)	7 (41.2%)	6 (54.5%)	0.67	-
	Adequate	4 (21.10%)	6 (35.30%)	4 (36.40%)		
	Excess	3 (15.80%)	4 (23.50%)	1 (9.10%)		

Total kcal/day adequacy to DRI girls	Deficit	6 (54.50%)	6 (50.00%)	8 (50.00%)	0.86	-
	Adequate	3 (27.30%)	5 (41.70%)	7 (43.80%)		
	Excess	2 (18.20%)	1 (8.30%)	1 (6.30%)		
kcal/kg/day adequacy to DRI boys	Deficit	13 (68.40%)	13 (76.50%)	8 (72.70%)	0.48	-
	Adequate	2 (10.50%)	2 (11.8%)	3 (27.30%)		
	Excess	4 (21.10%)	2 (11.80%)	0 (0%)		
kcal/kg/day adequacy to DRI girls	Deficit	9 (81.80%)	5 (41.70%)	12 (75.00%)	0.22	-
	Adequate	2 (18.20%)	5 (41.70%)	3 (18.80%)		
	Excess	0 (0%)	2 (16.7%)	1 (6.30%)		
Protein AMDR adequacy to DRI	Deficit	0 (0%)	0 (0%)	0 (0%)	-	-
	Adequate	30 (100%)	29 (100%)	27 (100%)		
	Excess	0 (0%)	0 (0%)	0 (0%)		
Grams of protein/kg/day adequacy to FAO recommendations	Deficit	0 (0%)	0 (0%)	0 (0%)	-	-
	Adequate	0 (0%)	0 (0%)	0 (0%)		
	Excess	30 (100%)	29 (100%)	27 (100%)		
CHs AMDR adequacy to DRI	Deficit	11 (36.70%)	19 (65.50%)	15 (55.60%)	0.079	-
	Adequate	19 (63.30%)	10 (34.50%)	12 (44.40%)		
	Excess	0 (0%)	0 (0%)	0 (0%)		
Simple sugars AMDR adequacy to DRI	Adequate	23 (76.70%)	27 (93.10%)	19 (70.40%)	0.085	-
	Excess	7 (23.30%)	2 (6.90%)	8 (29.60%)		
Lipids AMDR adequacy to DRI	Deficit	0 (0%)	0 (0%)	0 (0%)	0.43	-
	Adequate	5 (16.70%)	2 (6.90%)	5 (18.50%)		
	Excess	25 (83.30%)	27 (93.10%)	22 (81.50%)		
Linoleic acid adequacy to DRI	Deficit	27 (90.00%)	21 (72.40%)	23 (85.20%)	0.16	-
	Adequate	0 (0%)	0 (0%)	1 (3.70%)		
	Excess	3 (10.00%)	8 (27.60%)	3 (11.10%)		
Linolenic acid adequacy to DRI	Deficit	24 (80.00%)	20 (69.00%)	24 (88.90%)	0.30	-
	Adequate	0 (0%)	1 (3.40%)	0 (0%)		
	Excess	6 (20.00%)	8 (27.60%)	3 (11.10%)		
n-6 PUFAs AMDR adequacy to DRI	Deficit	29 (96.70%)	26 (89.70%)	23 (85.20%)	0.31	-
	Adequate	1 (3.30%)	3 (10.30%)	4 (14.80%)		
	Excess	0 (0%)	0 (0%)	0 (0%)		
n-3 PUFAs AMDR adequacy to DRI	Deficit	26 (86.70%)	26 (89.70%)	24 (88.90%)	0.82	-
	Adequate	4 (13.30%)	2 (6.90%)	3 (11.10%)		
	Excess	0 (0%)	1 (3.40%)	0 (0%)		

Parametrically distributed data are presented as mean ± SD and categorical data as n (%). [1] *p*-values are comparisons between COGNIS groups. [2] *p*-values for overall differences between study groups adjusted by maternal age, parents' educational level, and socioeconomic status through a multivariate analysis. ANOVA was carried out for normally distributed variables, and Chi-Square or Fisher's exact test for categorical variables. Values which do not share the same suffix (ab) are significantly different in a Bonferroni post-hoc test. Bold: *p*-values < 0.05. Total kcal is the total energy intake from the diet per day. Total Daily Energy Expenditure (TDEE) was calculated using the following equation: kcal/day (boys) = 88.5 – (61.9 x age [y]) + PA x [(26.7 x weight [kg]) + (903 x height [m])] + 20 and kcal/day (girls) = 135.3 – (30.8 x age [y]) + PA x [(10.0 x weight [kg]) + (934 x height [m])] + 20 (3-8 years old DRIs); PA: physical activity. Calculated kcal was obtained using this equation: Calculated kcal = (proteins intake x 4) + (carbohydrates intake x 4) + (lipids intake x 9). Kcal/kg/day was calculated by dividing Calculated kcal by the weight. Adj: adjusted; AMDR: Acceptable Macronutrient Distribution Ranges; ARA: arachidonic acid; BF: breastfeeding; CHs: carbohydrates; DHA: docosahexaenoic acid; DPA: docosapentaenoic acid; DRI: dietary reference

intake; EF: experimental infant formula; EPA: eicosapentaenoic acid; FAO: the Food and Agriculture Organization of the United Nations (38); kcal: kilocalories; MUFAs: Monounsaturated fatty acids; n-3 PUFAs: omega 3 polyunsaturated fatty acids; n-6 PUFAs: omega 6 polyunsaturated fatty acids; SF: standard infant formula; SFAs: Saturated Fatty Acids; TDEE: Total Daily Energy Expenditure. *Simple sugars AMDR has not been determined. The values were classified as adequate or excess according to the maximal intake level, ≤25% of total daily energy intake (40).
